# Six-lncRNA Immune Prognostic Signature for Cervical Cancer

**DOI:** 10.3389/fgene.2020.533628

**Published:** 2020-10-14

**Authors:** Qian Chen, Lang Hu, Dongping Huang, Kaihua Chen, Xiaoqiang Qiu, Bingqing Qiu

**Affiliations:** ^1^Department of Research, Guangxi Medical University Cancer Hospital, Nanning, China; ^2^Guangxi Medical University Cancer Hospital, Nanning, China; ^3^Department of Nutrition, School of Public Health, Guangxi Medical University, Nanning, China; ^4^Department of Radiation Oncology, Guangxi Medical University Cancer Hospital, Nanning, China; ^5^Department of Epidemiology, School of Public Health, Guangxi Medical University, Nanning, China; ^6^Department of Nuclear Medicine, Guangxi Medical University Cancer Hospital, Nanning, China

**Keywords:** TCGA, immune prognostic signature, lncRNA, cervical cancer, GSEA

## Abstract

**Background:**

This study searched for immune-related long noncoding RNAs (lncRNAs) to predict the prognosis of patients with cervical cancer.

**Method:**

We obtained immunologically relevant lncRNA expression profiles and clinical follow-up data from cervical cancer patients from The Cancer Genome Atlas database and the Molecular Signatures Database. Cervical cancer patients were randomly divided into a training group, testing group and combined group. The immune prognostic signature was constructed by Least Absolute Shrinkage and Selection Operator Cox regression, prognosis was analyzed by Kaplan–Meier curves between different groups, and the accuracy of the prognostic model was assessed by receiver operating characteristic-area under the curve (ROC-AUC) analysis.

**Results:**

A six-lncRNA immune prognostic signature (LIPS) was constructed to predict the prognosis of cervical cancer. The six lncRNAs are as follows: AC009065.8, LINC01871, MIR210HG, GEMIN7-AS1, GAS5-AS1, and DLEU1. A ROC-AUC analysis indicated that the model could predict the prognosis of cervical cancer patients in different subgroups. A Kaplan–Meier analysis showed that patients with high risk scores had a poor prognosis; these results were equally meaningful in the subgroup analyses. Risk scores differed depending on the clinical pathology and tumor grade and were independent risk factors for cervical cancer prognosis. Gene set enrichment analysis revealed an association between the LIPS and the immune response, Wnt signaling pathway, and TGF beta signaling pathway.

**Conclusion:**

Our study shows that the six-LIPS can predict the prognosis of cervical cancer and contribute to decisions regarding the immunotherapeutic strategy.

## Introduction

Cervical cancer is one of the main causes of death in females. Although screening and vaccination programs have been expanded, the number of new cases of cervical cancer has continued to increase, which means that cervical cancer is a major public health concern (Arbyn et al., 2020). Mortality rates in low-income countries and regions are vastly different from those in developed countries, with an 18-fold difference in mortality and 85% of deaths occurring in underdeveloped countries due to limited treatment options and economic and cultural factors (Small et al., 2017). At present, the conventional treatment of cervical cancer includes radiotherapy, chemotherapy and surgery, but patients at advanced stages are prone to developing radiotherapy and chemotherapy resistance (Seol et al., 2014). Therefore, it is necessary to identify new prognostic markers and treatment options for cervical cancer to improve the survival of cervical cancer patients.

The immune cells in the tumor microenvironment include B cells, CD8+ T cells, CD4+ T cells, and macrophages, among others. The response of cervical cancer to the immune system affects tumor progression and treatment (Chen R. et al., 2019). Immunotherapy is one of the most promising tools that may be used in future cervical cancer treatments. Immunotherapies consisting of anti-CTLA4 and anti-PD1 drugs have demonstrated efficacy in oropharyngeal cancer and cervical cancer (Dorta-Estremera et al., 2019; Grywalska et al., 2019). The combination of the immune checkpoint blockade PD1 and HPV16 E6/E7-targeted therapy can induce better anti-tumor effects (Dorta-Estremera et al., 2019; Zhen et al., 2019). Immunological checkpoint inhibitors can treat advanced chemotherapy-resistant cervical cancer (Baettig et al., 2019). Cytokines secreted by immune cells in the microenvironment inhibit the development of cervical cancer (Chauhan et al., 2019). The multi-immune infiltration cell signature in the tumor microenvironment has been shown to predict the prognosis of cervical cancer (Wang J. et al., 2019).

Long noncoding RNAs (lncRNAs) are defined as non-protein coding RNAs with lengths exceeding 200 nucleotides (Kapranov et al., 2007). lncRNAs have been reported to affect the occurrence and development of tumors. For example, a recent study showed that GAS5-AS1 inhibits the proliferation, migration and invasion of cervical cancer cells (Wang X. et al., 2019). LINC01535 was found to be elevated in cervical cancer tissue, is associated with a poor prognosis in cervical cancer and promotes the progression of cervical cancer via miR-214 EZH2 (Song et al., 2019). lncRNAs have also been found to regulate tumor immune responses. In diffuse large B cell lymphoma, the lncRNA SNHG14 promotes tumor progression and immune escape by regulating the PD-1/PD-L1 checkpoints (Zhao et al., 2019). In gastric cancer, the lncRNA HOTTIP has been reported to promote IL-6 expression, inhibit T cell proliferation and promote immune escape in gastric cancer cells (Shang et al., 2019). UCA1 (another lncRNA) expression is elevated in gastric cancer, and UCA1 can inhibit miR-214 expression and upregulate PDL1 expression, thus contributing to immune escape in gastric cancer cells (Wang C. J. et al., 2019). In addition, another study found that lncRNAs could be used as prognostic markers for tumor (Yao et al., 2019). In colon cancer, a five-lncRNA signature was reported to predict the survival of patients after patients were divided into high and low risk score groups, and high risk scores were associated with a poor prognosis (Lv et al., 2019). A recent study showed that the researchers constructed a ten-lncRNA signature to predict the 1-, 3-, and 5-year survival outcomes of ovarian cancer patients through Least Absolute Shrinkage and Selection Operator (LASSO) regression (Xu L. et al., 2019). In addition, several other methods for cancer prediction based on gene signatures and methylation patterns have been applied using LASSO regression (Sun et al., 2020; Zhang et al., 2020). However, there is no information on the prognostic value of immune-related lncRNAs in cervical cancer.

In this study, we obtained expression profiles and clinical data from patients with cervical cancer in The Cancer Genome Atlas (TCGA) database and extracted multiple immune-related lncRNAs from the Molecular Signatures Database v7.1 (MSigDB). Then, a lncRNA immune prognostic signature (LIPS) was constructed by LASSO Cox regression to predict the prognosis of patients with cervical cancer, we used six lncRNA to construct this signature, and the accuracy of the model was verified in different datasets to provide new insight into the prediction of prognosis and immunotherapy responses in cervical cancer.

## Materials and Methods

### Data Download

We downloaded clinical follow-up data and mRNA and lncRNA expression data from 307 patients with cervical cancer from the Genomic Data Commons website^1^ and filtered mRNAs and lncRNAs with expression values < 0.5 (Cancer Genome Atlas Research Network, Weinstein et al., 2013; Hernandez-Vargas et al., 2020). The inclusion criterion was patients who were followed up for more than 30 days, and a total of 273 patients were included in the study. The patients were randomly divided into a training group (*n* = 137) and a testing group (*n* = 136) at a ratio of 1:1.

### Identification of Immune-Related lncRNAs

We downloaded immune-related mRNAs from the MSigDB v7.1 (https://www.gsea-msigdb.org. Immune system process M13664, Immune response M19817) and extracted the expression profiles of cervical cancer patients (Subramanian et al., 2005). A cohort of immune-related lncRNAs were identified according to Pearson correlation analysis between the immune-related mRNAs and lncRNAs expression level in samples (| ρ| > 0.5, *P* < 0.05).

### Construction of the lncRNA Immune Prognostic Signature

We constructed a prognostic model of multiple lncRNA signatures by performing a LASSO Cox regression analysis of prognostic-associated immune lncRNAs using the “glmnet” package in R software. The *lambda.1se*, a penalty parameter used to prevent overfitting effects of the model was selected using 1000 times ten-fold cross validation. The lncRNA signature can be used to predict the risk score as follows:

$$Rscore=\sum {\text{SRNA}}_{\text{i}}\times {\text{Exp}}_{\text{lncRNAi}}$$

where RNA_*i*_ is the coefficient and Exp_*lncRNAi*_ is the lncRNA expression. Patients were divided into low-risk and high-risk groups according to the median risk score. The Kaplan–Meier (KM) method was used to analyze overall survival in the two groups. The accuracy of the multi-lncRNA signature for prognosis prediction was analyzed based on the receiver operating characteristic (ROC) method. The stability of the prediction model was also verified in the testing and combined groups (training group + testing group).

### Gene Set Enrichment Analysis and Immune Infiltration Analysis

Data from cervical cancer patients on immune cell infiltration were downloaded from the Tumor IMmune Estimation Resource (TIMER) database^2^, and the relationship between the risk score of the LIPS and the immune microenvironment was analyzed by Spearman’s correlation. Gene set enrichment analysis (GSEA) was used to analyze the functional enrichment of the high-risk and low-risk groups.

### Statistical Analysis

The LIPS was revealed as an independent prognostic risk factor for cervical cancer based on the univariate and multivariate Cox models. The relationship between the risk score and clinical indicators was examined by the chi-square test and Fisher’s exact test. The accuracy of the LIPS model when predicting cervical cancer was assessed by the area under the curve-receiver operating characteristic (AUC-ROC). Principal component analysis (PCA) was used to investigate the distribution of patients with different risk scores. The statistical analysis was performed using the R environment and Bioconductor packages (Version 3.5.5), and all statistical tests were two-sided and *p* value < 0.05 was considered to be statistically significant.

## Results

### Identification of Immune-Related lncRNAs

**FIGURE 1:**
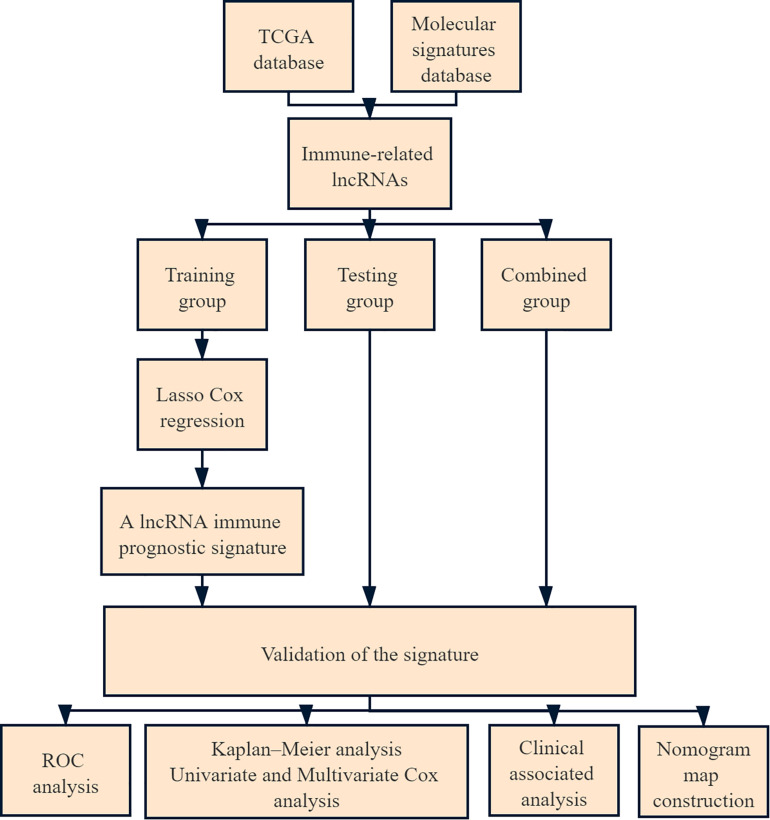
Flowchart of the analysis.

A total of 273 patients with cervical cancer were enrolled in the current study. Of these, 137 were in the training group, 136 were in the testing group, and 273 were in the combined group. The flow chart of this study is shown in Figure 1. We extracted 332 immune-related genes from the MSigDB and immune-related gene expression profiles from cervical cancer patients. Pearson’s correlation analysis was performed to analyze lncRNA expression in cervical cancer patients, with |ρ| > 0.5 and *p* value < 0.05 as the cut-offs. A total of 266 immune-related lncRNAs were found.

### Construction of the lncRNA Immune Prognostic Signature

**FIGURE 2:**
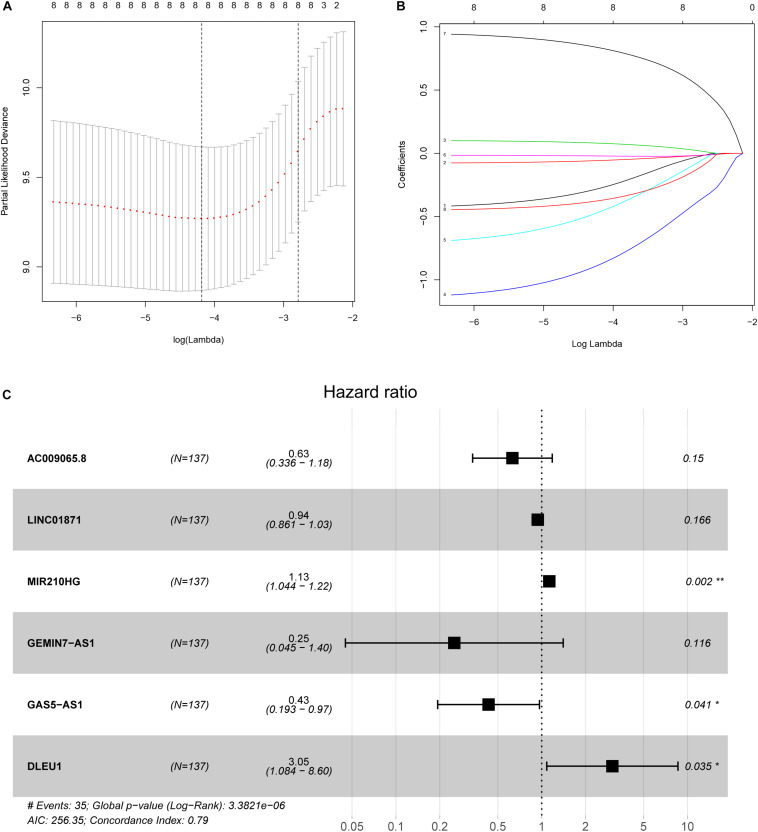
Ten-fold cross-validation for tuning parameter selection and a forest map. **(A)** Plots of the ten-fold cross-validation error rates. **(B)** LASSO coefficient profiles of the eight immune-related lncRNAs. **(C)** Forest map of the six prognostic lncRNAs by multivariate Cox regression.

**FIGURE 3:**
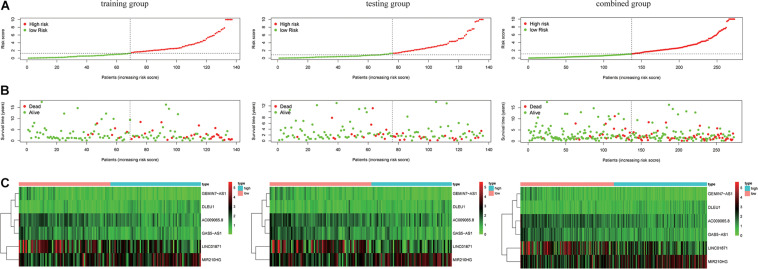
Risk score of the LIPS in the three groups. **(A)** Distribution of patients with different risk scores in the training group, testing group, and combined group. **(B)** Survival status of patients with different risk scores in the training group, testing group, and combined group. **(C)** Heatmap of the three-gene signature in the training group, testing group, and combined group.

**FIGURE 4:**
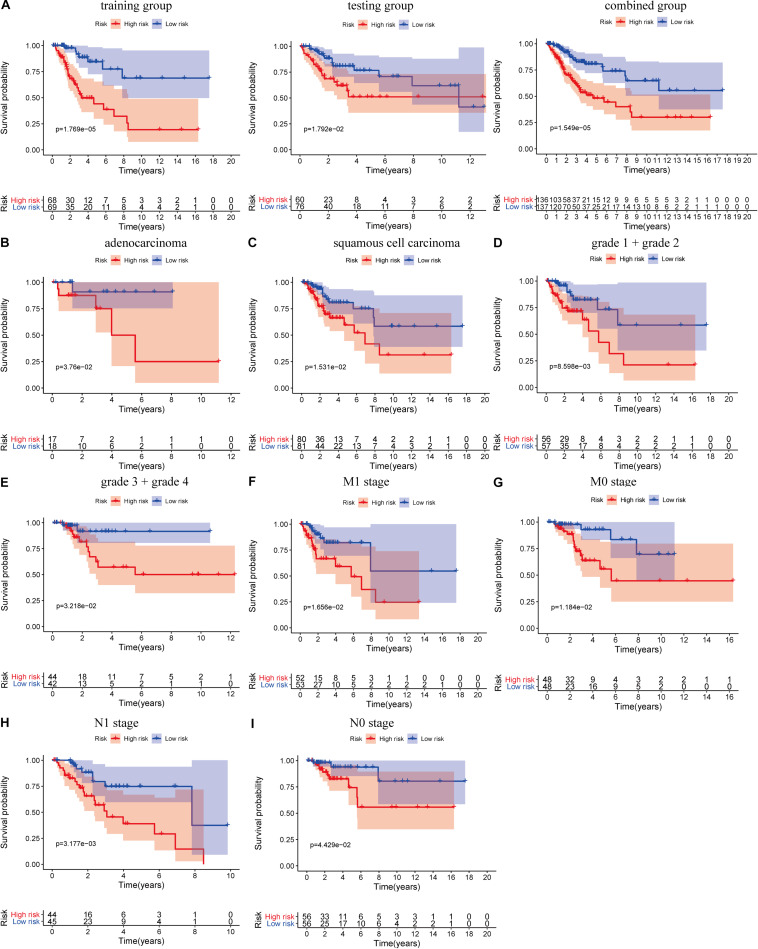
Prognostic significance and subgroup analysis of the LIPS. **(A)** Survival analysis of patients in the high and low risk score groups in the training group, testing group, and combined group. Subgroup analysis of the LIPS for **(B)** adenocarcinoma; **(C)** squamous cell carcinoma; **(D)** grade 1 + grade 2; **(E)** grade 3 + grade 4; **(F)** M1 stage; **(G)** M0 stage; **(H)** N1 stage; and **(I)** N0 stage.

In the training group, we used LASSO Cox regression to construct a multi-LIPS prognostic model using immunologically relevant lncRNAs to predict the prognosis of patients with cervical cancer with 1000 times ten-fold cross validation (Figure 2). The results showed that the prognostic model consisting of 6 lncRNAs could predict prognosis, and the following formula was used to calculate the risk score:

$$\begin{array}{c} Rscore=\left(-0.46211\times {\text{Exp}}_{\text{AC}009065.8}\right)+\\ \left(-0.06197\times {\text{Exp}}_{\text{LINC}01871}\right)+\left(0.120252\times {\text{Exp}}_{\mathrm{MIR210}\text{HG}}\right)+\\ \left(-1.38062\times {\text{Exp}}_{\mathrm{GEMIN7}-\mathrm{AS1}}\right)+\left(-0.83949\times {\text{Exp}}_{\mathrm{GAS5}-\mathrm{AS1}}\right)+\\ \left(1.116163\times {\text{Exp}}_{\mathrm{DLEU1}}\right) \end{array}$$

where Exp_*lncRNAi*_ is the lncRNA expression. According to the median risk score, the patients were divided into a high risk score group and a low risk score group (Figure 3). The results showed that in the training group, testing group and combined group, the 5-year survival rate of patients with a high risk score was lower than that of patients with a low risk score (Figure 4A). We then performed a subgroup analysis of the survival of patients with different ages, pathological types, grades, and T, M, N stages. The results showed that in the subtypes pathological type, grade, M, and N stages, the overall survival of cervical cancer patients in the high risk score group was shorter than that of cervical cancer patients in the low risk score group (Figures 4B–I).

### Prediction Accuracy of the LIPS

**FIGURE 5:**
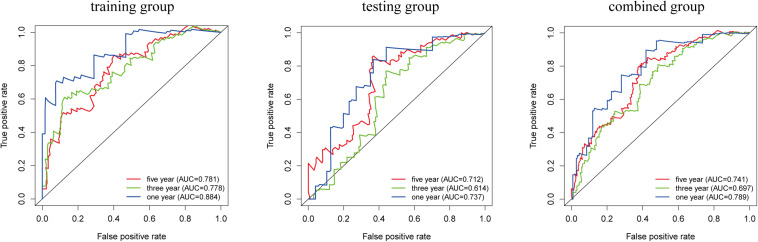
Prognostic value verification of the LIPS by ROC analysis in the training group, testing group and combined group.

We analyzed the accuracy of the LIPS to predict the prognosis of cervical cancer by ROC curve analysis (Figure 5). In the training group, the AUC-ROC value for 1-year survival was 0.884, the AUC-ROC value for 3-year survival was 0.778, and the AUC-ROC value for 5-year survival was 0.781. In the testing group, the AUC-ROC value for 1-year survival was 0.737, the AUC-ROC value for 3-year survival was 0.614, and the AUC-ROC value for 5-year survival was 0.712. In the combined group, the AUC-ROC value for 1-year survival was 0.789, the AUC-ROC value for 3-year survival was 0.697, and the AUC-ROC value for 5-year survival was 0.741. These results show that the LIPS can be a good indicator of the prognosis of cervical cancer.

### The LIPS Is an Independent Risk Factor for Cervical Cancer Prognosis

**FIGURE 6:**
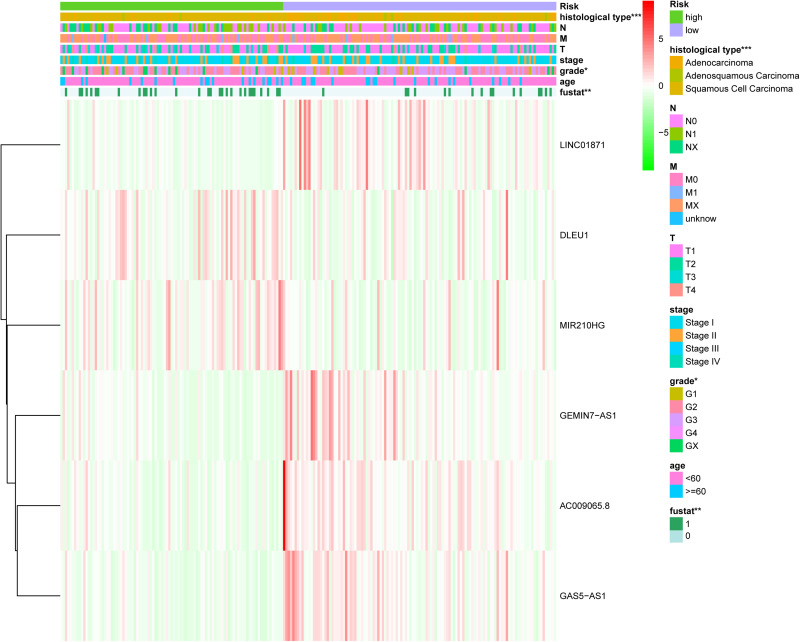
Relationship between the risk score and clinical significance. (*p* value*** < 0.001, *p* value** < 0.01, and *p* value* < 0.05).

**FIGURE 7:**
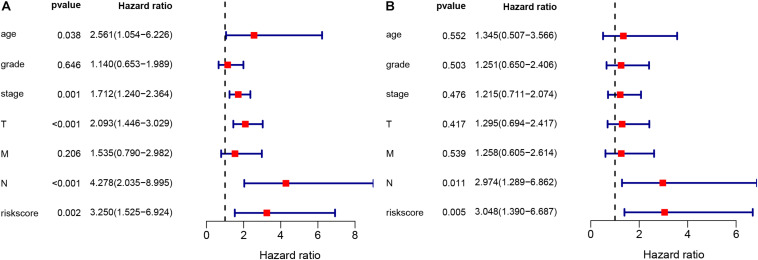
Univariate and multivariate Cox analyses of cervical cancer. **(A)** Univariate analysis. **(B)** Multivariate analysis.

**FIGURE 8:**
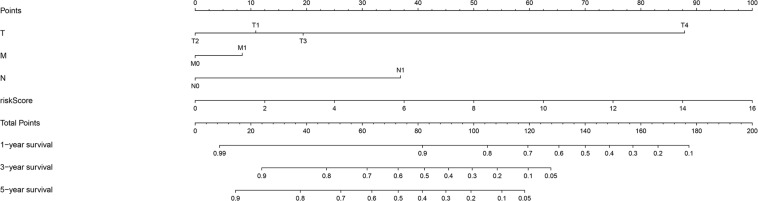
Nomogram used to predict prognosis in patients with cervical cancer at 1, 3, and 5 years.

The chi-square test and Fisher’s exact test showed that the risk score of the LIPS differed according to clinicopathological features and tumor grades (Figure 6). To determine whether the LIPS could be used as an independent risk factor for cervical cancer, we performed univariate and multivariate Cox analyses (Figure 7). The covariates included age, grade, T, M, N stages, and risk score. Univariate Cox regression analysis showed that age, stage, T, N, and risk score correlated with cervical cancer prognosis (*p* < 0.05). However, subsequent multivariate Cox analysis showed that the N (HR = 2.974, 95% CI = 1.289–6.862, and *p* = 0.011) and LIPS (HR = 3.048, 95% CI = 1.390–6.687, and *p* = 0.005) were independent risk factors for cervical cancer prognosis. We constructed nomogram maps to predict 1-, 3-, and 5-year survival in patients with cervical cancer using T, M, N, and risk score (Figure 8). A high score indicated poor clinical outcomes.

### Immune Infiltration and Pathway Analysis

**FIGURE 9:**
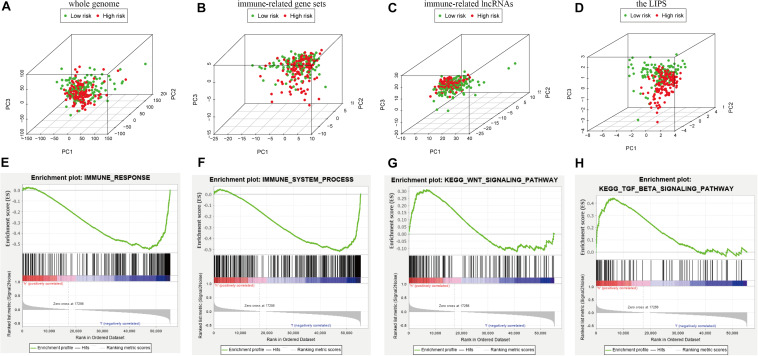
Patients with high and low risk scores have different immune statuses. PCA maps show the distribution of patients based on the **(A)** whole genome; **(B)** immune-related gene sets; **(C)** immune-related lncRNAs; and **(D)** the LIPS. The separation of the red and green dots becomes stronger when take only signature lncRNA. GSEA showed significant enrichment of the immune response **(E,F)** and tumor-related signaling pathways **(G,H)**.

**FIGURE 10:**
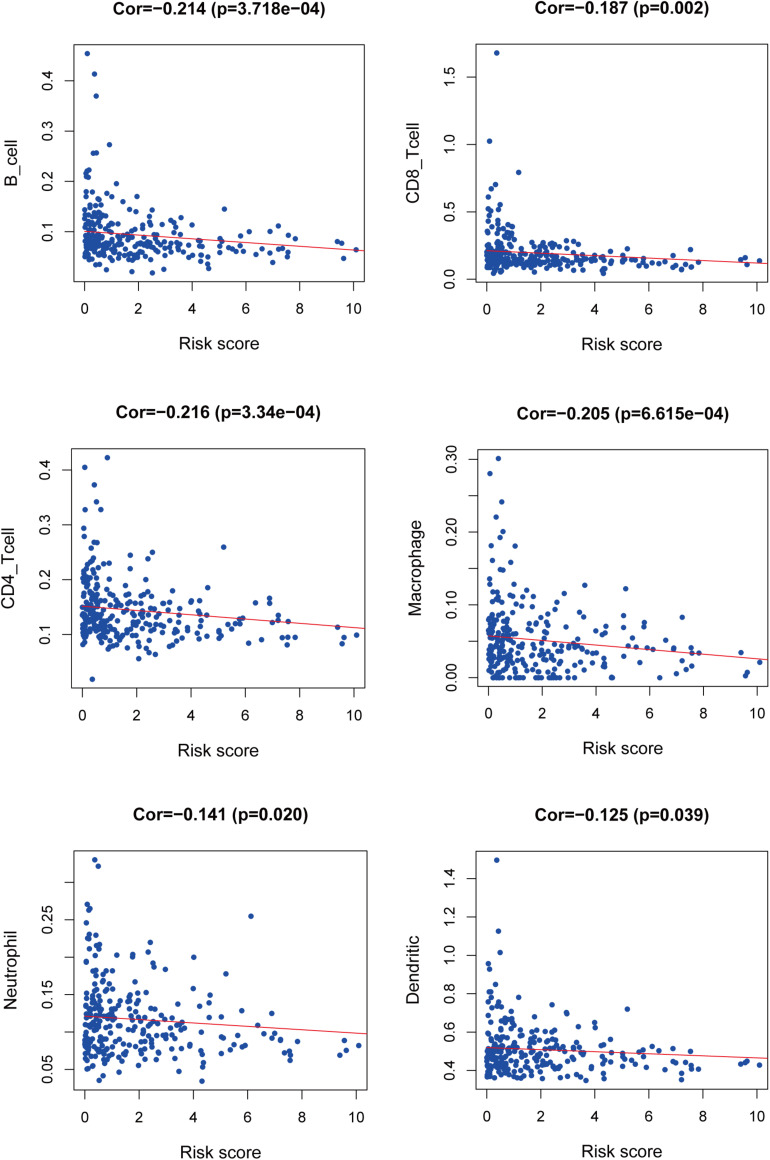
Relationship between the risk score and immune infiltration.

We used PCA maps to visualize the distribution of patients based on the whole genome, immune-related gene sets, immune-related lncRNAs, and the LIPS. The results showed that the LIPS was the best for patients (Figures 9A–D). Patients with high and low risk scores were distributed in different quadrants. GSEA showed that the functional pathways involved are mainly the immune response and tumor-related signaling pathways, such as the immune response signaling pathway, the Wnt signaling pathway, and the TGF beta signaling pathway (Figures 9E–H). By investigating a correlation between the risk score and immune cells in cervical cancer patients, we found that patients with low risk scores had high infiltration levels of B cells, CD8+ T cells, CD4+ T cells, macrophages, neutrophils, and dendritic cells in the immune microenvironment (Figure 10). These results indicate that patients with high and low risk scores are in different immune states.

## Discussion

The immune microenvironment affects the progression, metastasis, treatment and prognosis of cervical cancer. lncRNAs have been found to affect the tumor immune response and immune cell infiltration to affect tumor development. In colorectal cancer, SATB2-AS1 can affect the density of immune cells and the secretion of TH1-type chemokines (Xu M. et al., 2019). Lnc-INSR was found to regulate the differentiation of Treg cells to inhibit the immune microenvironment and thereby promote tumor growth (Wang et al., 2018b). However, immune-related lncRNA-based tools for the prognosis of cervical cancer are lacking.

In this study, we divided 273 patients with cervical cancer into a training group, testing group, and combined group. A total of 332 immune-related genes and 266 immune-related lncRNAs were revealed by the MSigDB. In the training group, we constructed a LIPS by LASSO Cox regression that includes AC009065.8, LINC01871, MIR210HG, GEMIN7-AS1, GAS5-AS1, and DLEU1. In the three groups, the overall survival of patients in the high risk score group was shorter than that of patients in the low risk score group. The prognostic analysis showed that the subgroups based on different pathological types, grades, M, and N were equally meaningful. ROC analysis showed that the LIPS was accurate in predicting the prognosis of cervical cancer in all three groups. Next, a chi-square test and Fisher’s exact test revealed that the LIPS differed according to pathological features and tumor grade. The multivariate Cox analysis showed that the immune-related lncRNA signature was an independent risk factor for cervical cancer prognosis. Finally, we constructed a nomogram map to predict the survival of patients with cervical cancer at 1, 3, and 5 years. The risk scores represent the clinical outcomes. Patients with high risk scores by LIPS had a poor prognosis. PCA showed that the LIPS could differentiate patients according to their immune status. These results demonstrate that the LIPS can be a good indicator of cervical cancer prognosis compared with the predictive power of existing signatures reported in recent studies (Table 1; Wu et al., 2019; Cheng et al., 2020; Liu et al., 2020a, b, c). Of these six lncRNAs, MIR210HG is a common lncRNA that is highly expressed in a variety of tumors. In the 373 colon cancer patients, the 5-year survival rate of patients in the MIR210HG high expression group was lower than the 5-year survival rate of patients in the MIR210HG low expression group (Ruan et al., 2019). In invasive breast cancer, MIR210HG is elevated in cancer tissues, and high MIR210HG expression is positively associated with a poor prognosis (Li et al., 2019). From the GSE30219 dataset, it was found that MIR210HG was upregulated in non-small cell lung cancer, the overall survival rate of the MIR210HG high expression group was lower than that of the MIR210HG low expression group, and MIR210HG could promote tumor cell proliferation and migration (Kang et al., 2019). GAS5-AS1 was found to affect the progression of different tumors. Wang X. et al. (2019) found that GAS5-AS1 is downregulated in cervical cancer tissues, inhibiting the biological function of cervical cancer cells, and that the downregulation of GAS5-AS1 is associated with a poor prognosis in cervical cancer. In hepatocellular carcinoma, GAS5-AS1 is downregulated in tissues and blood samples, and patients with low GAS5-AS1 expression have a poor prognosis (Wang et al., 2018a). Thus, GAS5-AS1 can be considered a diagnostic and prognostic marker for hepatocellular carcinoma. Another study found that GAS5-AS1 is an inhibitor of non-small cell lung cancer, downregulated in lung cancer, and inhibits the migration and invasion of lung cancer cells and epithelial-mesenchymal transition (EMT; Wu et al., 2016). DLEU1 has also been reported to be involved in the prognosis of several tumors. In osteosarcoma, DLEU1 is upregulated in tumor tissues, and CCK-8 experiments revealed that DLEU1 promotes cell proliferation (Chen X. et al., 2019). Colorectal cancer patients with high DLEU1 expression have a low survival rate and a poor prognosis. The inhibition of DLEU1 expression reduces the proliferation, migration, and invasion of colorectal cancer cells (Liu et al., 2018). Li et al. found that high DLEU1 expression was associated with tumor size, pathological stage, and lymph node metastasis. The KM analysis showed that high levels of DLEU1 were associated with a poor prognosis, and Cox regression analysis showed that DLEU1 is an independent prognostic risk factor for gastric cancer; experiments have also shown that DLEU1 is silenced to induce tumor cell apoptosis (Li et al., 2018). As we have discussed, these lncRNAs play a key role in tumors where they regulate their functions to exhibit anti-tumor effects. Therefore, lncRNA-based therapy is a very promising and important method that may be used in the future, especially in nucleic acid-based therapeutics. It is expected that new targeted drugs will be developed to inhibit tumor proliferation and metastasis and improve patient survival. In addition, the importance of AC009065.8, LINC01871, and GEMIN7-AS1 in cervical cancer is rarely reported in the literature, and thus, we will focus on these three lncRNAs in future research.

**TABLE 1:** Comparison of studies of existing signatures for cervical cancer.

Databases	Methods	Signature	Symbols	AUC value	References
TCGA	LASSO cox analyses	Two-mRNAs	CD1C and CD6	0.72	Liu et al.
TCGA	Multivariate cox analyses	Three-mRNAs	FLT1, GAPDH, EZH2	0.698	Liu et al.
TCGA	Multivariate cox analyses	Three-mRNAs	ITGA5, HHEX, and S1PR4	0.749	Liu et al.
TCGA	Multivariate cox analyses	Two-lncRNAs	ILF3-AS1 and RASA4CP	0.607	Wu et al.
TCGA	Multivariate cox analyses	Six-lncRNAs	LINC00619, FGF13-AS1, EMX2OS, WT1-AS, C9orf147, and LINC00908	0.65	Cheng et al.

We performed an enrichment analysis of the LIPS with GSEA, and the results showed that the LIPS was enriched in the immune response, Wnt signaling pathway, and TGF beta signaling pathway. Cervical cancer cells can react with the immune microenvironment to escape from the immune response and promote tumor development (Heusinkveld et al., 2011). Previous studies have shown that activation of the Wnt signaling pathway is the first step in carcinogenesis of the cervix (Uren et al., 2005). The Wnt signaling pathway regulates proliferation, migration, and chemoradiation tolerance in cervical cancer cells and is associated with poor clinical outcomes (Bahrami et al., 2017; Yang et al., 2018; Liu et al., 2020d). Drews et al. (2019) found that E6 degrades NHERF1 to regulate the Wnt signaling pathway and affect the progression of cervical cancer. The TGF beta signaling pathway is also a classic pathway that affects tumor progression. In the tumor microenvironment, cervical cancer-associated fibroblasts enhance the ability of cervical cancer cells to invade by secreting TGF-β (Nagura et al., 2015). Deng et al. (2019) revealed that CD36 binds to TGF-β to promote EMT, which leads to the migration and invasion of cervical cancer. Twist also regulates EMT via the TGF beta signaling pathway and promotes the migration of cervical cancer cells (Fan et al., 2015). Previous reports indicate that cervical cancer is in an immunosuppressed microenvironment with multiple immune escape strategies (Piersma, 2011). We found that patients with high risk scores had a poor prognosis and low levels of immune cell infiltration in the immune microenvironment. PCA revealed that cervical cancer patients with high and low risk scores are in different immune states. The above results show that six immune-related lncRNAs play a key role in the immune microenvironment.

Immunotherapy is currently one of the most promising treatments, and an increasing number of studies are focused on the relationship between the immune microenvironment and cervical cancer. Although we first constructed a LIPS to predict the prognosis of cervical cancer and showed good results with different datasets, our research still has some limitations. Therefore, we will continue to search for novel data and to perform experimental testing of lncRNAs that have been found. We need to establish cellular and animal models to validate these results using PCR, immunohistochemistry and western blot techniques. Currently, we are conducting cell-based experiments on multiple lncRNAs.

In summary, we constructed a LIPS to predict the prognosis of cervical cancer and validated the results by using different datasets. We also determined that the LIPS is an independent risk factor for cervical cancer. We hope to provide a new reference for the current prognostic assessment of cervical cancer and bring new insight into immunotherapy strategies.

## Data Availability Statement

The datasets presented in this study can be found in online repositories. Publicly available datasets were analyzed in this study. This data can be found here: https://portal.gdc.cancer.gov/.

## Author Contributions

All authors contributed to the article and approved the submitted version.

## Conflict of Interest

The authors declare that the research was conducted in the absence of any commercial or financial relationships that could be construed as a potential conflict of interest.

## Footnotes

1 https://portal.gdc.cancer.gov/

2 https://cistrome.shinyapps.io/timer/
